# The Evolving Microbiome from Pregnancy to Early Infancy: A Comprehensive Review

**DOI:** 10.3390/nu12010133

**Published:** 2020-01-02

**Authors:** María Dolores Mesa, Begoña Loureiro, Iris Iglesia, Sergi Fernandez Gonzalez, Elisa Llurba Olivé, Oscar García Algar, María José Solana, Mª Jesús Cabero Perez, Talia Sainz, Leopoldo Martinez, Diana Escuder-Vieco, Anna Parra-Llorca, María Sánchez-Campillo, Gerardo Rodriguez Martinez, Dolores Gómez Roig, Myriam Perez Gruz, Vicente Andreu-Fernández, Jordi Clotet, Sebastian Sailer, Isabel Iglesias-Platas, Jesús López-Herce, Rosa Aras, Carmen Pallás-Alonso, Miguel Saenz de Pipaon, Máximo Vento, María Gormaz, Elvira Larqué Daza, Cristina Calvo, Fernando Cabañas

**Affiliations:** 1 Department of Biochemistry and Molecular Biology II, Institute of Nutrition and Food Technology “José Mataix”, Biomedical Research Center, University of Granada, Parque Tecnológico de la Salud, Avenida del Conocimiento s/n, Armilla, 18100 Granada, Spain; 2 ibs.GRANADA, Instituto de Investigación Biosanitaria, Complejo Hospitalario Universitario de Granada, 18014 Granada, Spain; 3 Neonatology Unit, University Hospital Cruces, Biocruces Bizkaia Health Research Institute, 48903 Barakaldo, Spain; begona.loureirogonzalez@osakidetza.eus; 4 Growth, Exercise, Nutrition and Development (GENUD) Research Group, Universidad de Zaragoza, 50009 Zaragoza, Spain; iglesia@unizar.es; 5 Instituto de Investigación Sanitaria Aragón (IIS Aragón), 50009 Zaragoza, Spain; gerard@unizar.es; 6 BCNatal-Barcelona Center for Maternal-Fetal and Neonatal Medicine (Hospital Sant Joan de Deu and Hospital Clínic), 08028 Barcelona, Spain; sfernandezgo@sjdhospitalbarcelona.org (S.F.G.); ogarciaa@clinic.cat (O.G.A.); lgomezroig@hsjdbcn.org (D.G.R.); mperezc@sjdhospitalbarcelona.org (M.P.G.); viandreu@clinic.cat (V.A.-F.); jclotet@clinic.cat (J.C.); sebastiansailer34@gmail.com (S.S.); iiglesias@sjdhospitalbarcelona.org (I.I.-P.); 7 Institut de Recerca Sant Joan de Déu (IR-SJD), 08028 Barcelona, Spain; 8 Obstetrics and Gynecology Department, High Risk Unit, Sant Pau University Hospital, 08025 Barcelona, Spain; llurba@yahoo.es; 9 Women and Perinatal Health Research Group, Biomedical Research Institute Sant Pau (IIB-Sant Pau), Sant Pau University Hospital, 08041 Barcelona, Spain; 10 School of Medicine, Universitat Autònoma de Barcelona, 08193 Barcelona, Spain; 11 Neonatology Unit, Hospital Clinic-Maternitat, ICGON, BCNatal, Gran Via de les Corts Catalanes, 585, 08007 Barcelona, Spain; 12 Servicio de Cuidados Intensivos Pediátricos, Hospital General Universitario Gregorio Marañón, Departamento de Salud Pública y Materno infantil, Universidad Complutense de Madrid, 28040 Madrid, Spain; mjsolana@hotmail.com (M.J.S.); pielvi@hotmail.com (J.L.-H.); 13 Hospital Universitario Marqués de Valdecilla, Santander, 39008 Cantabria, Spain; mariajesuscabero@gmail.com; 14 Servicio de Pediatría, Enfermedades Infecciosas y Tropicales, Hospital La Paz, 28046 Madrid, Spain; tsainzcosta@gmail.com (T.S.); ccalvorey@gmail.com (C.C.); 15 Instituto de Investigación Hospital la Paz (IdiPAZ), 28029 Madrid, Spain; leopoldo.martinez@salud.madrid.org (L.M.); rosa.aras@hotmail.com (R.A.); 16 Red de investigación Traslacional en Infectología Pediátrica (RITIP), 28046 Madrid, Spain; 17 Servicio de Cirugía Pediátrica, Hospital La Paz, 28046 Madrid, Spain; 18 Donated Milk Bank, Health Research Institute i + 12, University Hospital 12 de Octubre, Universidad Complutense, 28040 Madrid, Spain; diana.e.vieco@gmail.com (D.E.-V.); kpallas.hdoc@gmail.com (C.P.-A.); 19 Neonatal Research Group, Health Research Institute La Fe, University and Polytechnic Hospital La Fe, 46026 Valencia, Spain; annaparrallorca@gmail.com (A.P.-L.); Maximo.Vento@uv.es (M.V.); mgormi@yahoo.es (M.G.); 20 Department of Physiology, Faculty of Biology, University of Murcia, 30100 Murcia, Spain; medit2011@gmail.com (M.S.-C.); elvirada@um.es (E.L.D.); 21 Department of Pediatrics, Faculty of Medicine, Hospital Clínico Universitario Lozano Blesa, 50009 Zaragoza, Spain; 22 Neonatology Unit, Hospital Sant Joan de Déu, Institut de Recerca Sant Joan de Déu, BCNatal, 08028 Barcelona, Spain; 23 Department of Neonatology La Paz University Hospital, 28046 Madrid, Spain; 24 European Network of Excellence for Pediatric Clinical Research, 27100 Bari, Italy; 25 Department of Paediatrics-Neonatology Quironsalud, Madrid University Hospital and Biomedical Research Foundation-IDIPAZ, La Paz University Hospital, 28046 Madrid, Spain; fernando.cabanas@quironsalud.es

**Keywords:** microbiome, pregnancy, fetus, placenta, newborn, infancy, critical illness, sepsis, allergy

## Abstract

Pregnancy induces a number of immunological, hormonal, and metabolic changes that are necessary for the mother to adapt her body to this new physiological situation. The microbiome of the mother, the placenta and the fetus influence the fetus growth and undoubtedly plays a major role in the adequate development of the newborn infant. Hence, the microbiome modulates the inflammatory mechanisms related to physiological and pathological processes that are involved in the perinatal progress through different mechanisms. The present review summarizes the actual knowledge related to physiological changes in the microbiota occurring in the mother, the fetus, and the child, both during neonatal period and beyond. In addition, we approach some specific pathological situations during the perinatal periods, as well as the influence of the type of delivery and feeding.

## 1. Introduction

Pregnancy induces a number of immunological, hormonal, and metabolic changes necessary for the normal development of the fetus and for a timely onset of labor and successful delivery [1]. It has been described that maternal microbiota influences prenatal and early postnatal offspring development and health outcomes [2,3]. There is a lack of consensus about the real nature of microbiome changes during pregnancy, since discrepant and unpredictable findings have been described [4,5,6]. These differences could be explained by the difference in gestational age, genetics, ethnicity, and environmental factors surrounding the participants included in those studies. Indeed, it has been described that maternal microbiota composition during pregnancy is related to maternal diet [7,8,9], and by pre-pregnancy weight and weight gain over the course of pregnancy [10,11,12,13]. Koren et al. described that the amounts of anti-inflammatory butyrate-producer commensal bacteria present in non-pregnant women gut microbiota decrease while bacteria associated with pro-inflammatory responses, such as *Proteobacteria*, increase during pregnancy [4]. Similarly, bacterial diversity tends to be reduced in vaginal microbiota during pregnancy while increasing vaginal *Streptococci* along with several specific *Lactobacilli* strains, which are thought to prevent the growth of pathogenic bacteria, as well as to help human digestion, and influence host innate and adaptive immune system responses [4,14]. Furthermore, the classical paradigm of the fetus as a sterile organism is under discussion, since a characteristic microbiome has been identified in the placenta, the amniotic fluid, and the fetus in healthy pregnancies [15,16]. However, this issue is under discussion. Perez-Muñoz et al. argued the weakness of evidence supporting the “in utero colonization hypothesis”, due to methodological difficulties, and concluded that current scientific evidence does not support the existence of microbiome within the healthy fetal milieu [17].

Gut microbiota influences the immune function [18], and thus may modulate the response through different microbial-derived metabolites, especially short-chain fatty acids (SCFAs) such as butyrate, acetate, or propionate [19]. These are the key drivers of T-cell subset proliferation and activity [19,20]. Gastrointestinal bacteria generate SCFAs after fermentation of complex dietary carbohydrates. These metabolites may have an influence both in the mother and in the newborn by down-regulation of pro-inflammatory responses at the specific sites where the allergens are located, which typically precedes asthma in childhood [21]. In addition, the may also influence bone marrow stimulation by reprogramming the immunological tone of the mammalian ecosystem [22].

Finally, it is important to consider that the discrepancies of the data obtained to date could be influenced by a number of factors such as the dietary pattern, the ethnicity, the geographic location, and the research methodology. The limitations of classical culturable methods have been improved with new molecular methods used to characterize the microbiota. However, these new methods have their own limitations, as reagent, laboratory contamination, and the inability to differentiate living and dead microorganisms. Indeed, recent research complements the study of microbiome with metabolomics and proteomic analysis in order to complete the whole metabolic picture of the microbiota and its metabolic status. Therefore, further studies are needed to confirm the evolution of microbiota during pregnancy and its influence in healthy and complicated labors and the newborn [23].

The present review summarizes the actual knowledge related to changes in maternal and fetal microbiota occurring during pregnancy, which may influence the newborn and infant development. In addition, changes in specific pathological infancy situations have also been revised.

## 2. Changes in the Microbiome during Pregnancy 

During pregnancy, the female body undergoes hormonal, metabolic, and immunological changes to preserve the health of both the mother and the offspring [1]. These changes alter the mother microbiota at different sites such as the gut, the vagina, and the oral cavity. However, published data are not consistent, since a number of factors might influence the microbiota profile such as the diet, antibiotic, or other supplement intakes, as well as the methodology of research. Therefore, a holistic approach is needed to understand all this information. 

### 2.1. Gut Microbiota 

**Figure 1:**
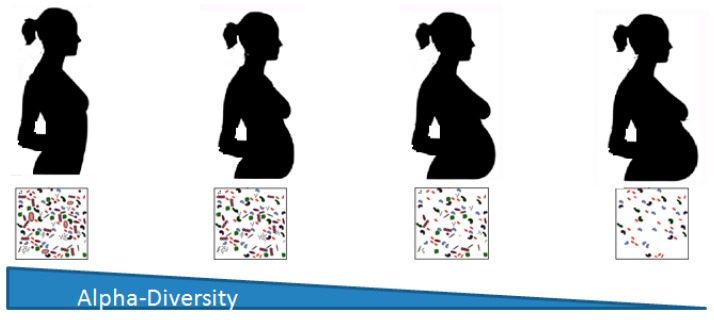
Alpha-diversity changes in gut microbiota during pregnancy.

The gut microbiota shifts substantially throughout the progression of the pregnancy and is characterized by reduced individual richness (alpha-diversity) (Figure 1), and increased inter-subject beta-diversity [4]. These changes are not related to, although they may be influenced by, the diet, antibiotic treatments, gestational diabetes, or pre-pregnancy body mass index, but are vital for a healthy pregnancy [4]. It has been suggested that other factors, such as the state of the host immune and endocrine systems, may actively contribute to the observed modifications [24]. During the first trimester, the gut microbiota pattern is similar in many aspects to that of healthy non-pregnant women, showing a predominance of *Firmicutes*, mainly *Clostridiales*, over *Bacteroidetes* [25]. Then, maternal gut microbiota declines in butyrate-producing bacteria, while *Bifidobacteria*, *Proteobacteria*, and lactic acid-producing bacteria increase from the first to the third trimester, when the microbiota resembles an unpredictably disease-associated dysbiosis that differs greatly among normal pregnancies [4]. Changes in the host immune system of the gastrointestinal mucosa together with metabolic hormonal changes may trigger a low-grade pro-inflammatory status that could facilitate an increased diffusion of glucose from the gut epithelium towards the lumen, and thus may induce weight gain while modifying the gut microbiota during normal pregnancies [26]. Indeed, changes in the microbiota may contribute to the evolution of this process. In addition, disruption of maternal gut microbiota during the third trimester [27] may affect host metabolism in order to provide an energy supply for the fetus [4,26]. Moreover, it has been reported that the gut microbiota during pregnancy is a critical determinant of offspring health [13,28], and that potentially determines the development of atopy and autoimmune phenotypes in the offspring [28]. However, the relationship among the immune system, the gut microbiota, and metabolism in pregnancy is unclear, and more research is needed to stablish final conclusions. 

### 2.2. Vaginal Microbiota

The composition of the vaginal microbiota is dynamic, corresponding with hormonal fluctuations throughout the woman’s reproductive life, and also during pregnancy. A number of protective lactic acid-producing *Lactobacillus* species dominates the healthy vaginal microbiota in most reproductive-age women. These bacteria protect against vaginal dysbiosis and inhibit opportunistic infections through the direct and indirect protective effects of *Lactobacillus* products, such as lactic acid and bacteriocin among others. Lactic acid decreases vaginal pH and thus inhibits a broad range of infections [29], can directly affect host immune functions, by inhibiting pro-inflammatory responses, and also help to release mediators from vaginal epithelial cells and stimulate antiviral response [30]. In addition, *Lactobacillus*-derived bacteriocins may inhibit pathogen growth [31]. The degree of protection varies according to the predominant *Lactobacillus* specie [30]. Vaginal dysbiosis is comprised of a wide array of strict and facultative anaerobes that correlate to increased risk of infection, diseases, and poor reproductive and obstetric outcomes [32]. 

During normal pregnancy, the composition of the vaginal microbiota changes as a function of gestational age, with an increase in the relative abundance for *Lactobacillus* spp., such as *L. crispatus*, *L. jensenii*, *L. gasserii*, *L. vaginalis*, and a decrease in anaerobe or strict anaerobe microbial species, such as *Atopobium*, *Prevotella*, *Sneathia*, *Gardenerella*, *Ruminococcaceae*, *Parvimonas*, *Mobilincus* [33]. Those authors reported for the first time, that the composition and stability of the vaginal microbiota of normal pregnant women is different from that of non-pregnant women. In fact, low risk pregnant women have more stable vaginal flora throughout the pregnancy than non-pregnant women. Normal changes in the vaginal flora during pregnancy are transitions to another *Lactobacillus* community, and this stability would protect against ascending infections through the genital tract. In addition, they reported that *Lactobacillus* communities vary depending on the ethnicity of the women [33]. Stout et al. [34] confirmed that vaginal microbiota richness and diversity remained stable during the first and second trimesters of gestation in pregnancies ended at term, whereas in woman with preterm born, the richness and diversity decreased early in pregnancy. Therefore, early pregnancy may be an important environment, modulating preterm delivery. A meta-analysis reported significant diversity differences in vaginal microbiomes in the first trimester, between women with term and preterm outcomes, indicating a potential diagnostic utility of microbiome-related biomarkers [35]. In addition, the increase of pathogens in the vagina is associated with complications of pregnancy, in particular with an increased risk of preterm birth and spontaneous abortion [6]. 

### 2.3. Oral Microbiota

An increase in the microbial load in the oral cavity during pregnancy has been described. It has been hypothesized that pregnancy creates a nutrient environment that is more favorable to some sensitive strains [36]. The presence of pathogenic bacteria *Porphyromonas gingivalis* and *Aggregatibacter actinomycotemcomitans* in gingival sulcus were significantly higher during early and middle stages of pregnancy compared to non-pregnant women [37]. The oral alpha-diversity index was higher in the third trimester compared to non-pregnant women, and this may be related to the increase of progesterone and estradiol. [38]. One underlying mechanism refers to estrogens being substituted for vitamin K in bacterial anaerobic respiration, especially for black-pigmented *Bacteroides* such as *Bacteroides melaninogenicus* and *Prevotella intermedia* [38].

### 2.4. Placental Microbiota and Fetal Colonization

The classical paradigm of fetal environment as a sterile harbor has traditionally explained that microbes, and thus microbiome, are acquired both vertically (from the mother) and horizontally (from other humans or from the environment) during and after birth. However, recent data have questioned the traditional accepted dogma of human microbiome acquisition, proposing that neither the placenta, the amniotic fluid, nor the fetus are sterile. 

Several findings using both culture and metagenomic techniques have suggested the presence of a low biomass microbial community in the healthy placenta [39,40,41,42,43]. The abundance of different species of *Lactobacillus*, *Propionibacterium*, and members of the *Enterobacteriaceae* family have been detected by DNA-based studies in placental tissue of pregnant women at term and it is under debate [16]. In addition, other authors have confirmed a distinct microbiota in both the placenta and amniotic fluid of healthy women at the time of elective C-section, characterized by low richness, low diversity, and the predominance of *Proteobacteria* [44]. Similarly, other studies have found microbes in amniotic fluid and umbilical cord blood in healthy asymptomatic women, as well as in those with pregnancy complications [45,46,47].

However, it is unclear where the fetal microbiota comes from, and when is the first fetal exposition. The presence of a different placental microbiota compared to the vagina raises the possibility that the infant may be first seeded in utero from other sources. Microorganism may pass through the placenta and colonize the fetus ascending from the vagina, from the oral cavity, from the urinary track, or from the intestinal lumen of the mother. These microorganisms may reach via the hematogenous route, the placenta, and then be transmitted to the fetus [48]. Some of those oral bacteria, such as *Fusobacterium nucleatum*, may be transmitted hematogenously during placentation by binding to the vascular endothelium, and modifying its permeability and the translation of other common commensals, such as *Escherichia coli* [49]. In addition, Franasiak et al. observed that *Flavobacterium* and *Lactobacillus* represent the majority of endometrial bacterium at the time of embryo transfer, supporting a new hypothesis of the endometrial environment participation [50].

Different studies have also detected microbiome in the first baby fecal sample, the meconium, supporting the in utero exposure to bacteria [51,52]. *Staphylococcus* has been reported as the most prevalent bacteria in meconium samples, followed by *Enterobacteriaceae*, *Enterococcus*, *Lactobacillus*, and *Bifidobacterium* even in infants born by C-section [52,53]. Modification in placental microbiota may be related with adverse pregnancy outcomes of pregnancy or symptoms of clinical infection [40]. 

On the contrary, Perez-Muñoz et al. [17] critically revised scientific evidence supporting both the “sterile womb” and “in utero colonization” hypotheses. These authors concluded that there is more evidence supporting a sterile womb environment. They suggest that methodological approaches, in which contamination is very easy at different steps and does not use appropriate controls, are responsible for the microorganism colonization described in utero. One well-controlled study compared oral, vaginal, and placenta samples with paired contamination controls. This study reported that when using molecular methods, placental samples were undistinguishable from their paired-contaminated samples. They concluded that while there were distinctive microbial signatures in oral and vaginal samples, they did not find a characteristic placental microbiota, evidencing a sterile environment [54]. Therefore, conclusions remain unachievable, and more studies are needed in this area. 

## 3. Changes in the Microbiome Related to the Type of Delivery

There is great controversy in the scientific community about the relationship of the meconium and infant gut microbiota profile, and the type of delivery. Microbiome studies on early infancy have demonstrated a significant influence of the mode of delivery on the microbiome composition, suggesting the likely association of the infant gut bacteria with maternal vaginal or skin microbiome habitats. A systematic review has concluded that the diversity and colonization pattern of the gut microbiota were significantly associated to the mode of delivery during the first three months of life, which is a critical period of life for immunological programming [55]. However, the observed differences disappear after 6 months of infants’ life, when solid foods are included in the diet [56]. It is important to clarify the influence of factors commonly accompanying C-section delivery on the microbiome, due to the potential influence on some non-communicable diseases, such as neonatal skin infection, asthma, allergies, obesity, inflammatory bowel disease, or type I diabetes mellitus [56,57]. 

Vaginally delivered newborn have shown bacterial communities resembling their own mother’s vaginal microbiota, dominated by *Lactobacillus*, *Prevotella*, or *Sneathia* spp. In contrast, C-section-born infants harbored bacterial communities similar to those found on the skin surface niche, dominated by *Staphylococcus*, *Corynebacterium*, and *Propionibacterium* spp. [58] or potentially pathogenic microbial communities such as *Klebsiella*, *Enterococcus*, and *Clostridium* [57]. Other authors have reported that *Bifidobacterium* [59] and *Bacteroides* [55] seem to be significantly more frequent in vaginally compared with C-section delivered infants, which were mainly colonized by *Clostridium* and *Lactobacillus* [55]. The high abundance of *Bifidobacterium* species in infants is considered to promote the maturation of the healthy immune system, while high presence of *Clostridium difficile* is considered as one of the major intra-hospital hazards of severe gastrointestinal infections during infancy [55]. Another study proposed that some species of *Propionibacterium* were most abundant in the meconium of vaginally delivered Chinese infants, whereas C-section-born children had higher amounts of *Bacillus licheniformis*. In addition, the diversity of the microbial composition was also higher in vaginal than in C-section deliveries, although no correlation with maternal microbiome was reported [60]. Similarly, a metagenomic analysis found a *Propionibacterium*-enriched meconium in vaginal delivery mothers, which may proceed from skin or fecal microbes through direct contact during the natural labor [61]. Therefore, there is no consensus regarding the most colonizable pattern of the first microbiota community in the first three days after birth, although it seems that according to phyla, vaginal deliveries are more related to *Actinobacteria* and *Bacteroidetes*, while C-section deliveries are more related to *Firmicutes*. In addition, it has also been suggested that the transfer of maternal vaginal microbes plays a minor role in seeding infant stool microbiota since the overlap of maternal vaginal microbiota and infant faecal microbiota is minimal, while the similarity between maternal rectal microbiota and infant microbiota was more pronounced [62]. 

The discrepances of the results obtained could be due to different factors associated to C-section delivery such as antibiotic administration, but also to breastfeeding, maternal obesity, gestational diabetes mellitus, and even the analytical methodology. In addition, the diversity from *Firmicutes* and *Bacteroides* colonization levels on infants gut microbiota may be influenced by geographical variation such as the latitude [63].

Some authors have proposed that the lower presence of *Bifidobacteria* and *Bacteroides*, and the abundance of *Clostridia* and *Lactobacillus*, in infants delivered by C-section could be explained by perinatal antibiotics administration [55]. Mothers delivering by C-section receive antibiotic prophylaxis before the beginning of surgery or, in some countries, after the cord clamping to minimize the direct exposure of the neonate to antibiotics [64]. In addition, Azad et al. determined that intrapartum antibiotics both in C-section and vaginal deliveries are associated with infant gut microbiota dysbiosis, although breastfeeding modifies some of these effects [65]. Nevertheless, Martinez et al. [66] performed antibiotic-free C-section delivery in mice and determined that these mice did not have the dynamic developmental gut microbiota changes observed in control natural born mice, evidencing the involvement of maternal vaginal bacteria in a proper metabolic development even in absence of antibiotics supporting the hypothesis of the antibiotic-modulated dysbiosis. It is worth to take into account that perinatal antibiotic administration may be associated with increased risk of developing morbidities such as asthma, allergies and obesity, which may be influenced by dysbiosis. In accordance, epidemiological data show that atopic diseases appear more often in infants born by C-section than after vaginal delivery [67,68]. 

Furthermore, bacterial richness and diversity were lower in the infant gut of babies born after elective C-section and higher in emergency C-section, suggesting that colonization may be affected differently in both situations. It is important to highlight that emergency C-section and vaginal delivery labor are frequently accompanied by rupture of fetal membranes, and exposing the fetus to maternal vaginal bacteria [65]. 

Importantly, C-section may decrease the colonization of milk-digested bacteria including the genus *Lactobacillus* in newborns during the first months of life [58]. In addition, the mode of delivery has a relevant impact on the microbiota composition of colostrums and milk [69,70], which also may be influenced by antibiotics administrated during C-section. It has been proposed that infants born by C-section lacked the early provision of breast milk essential to attain a proper gut microbiota that contains microbes such as *Lactobacilli* and *Bifidobacteria.* This could explain the higher colonization rates of these genera in vaginal compared to C-section-delivered infants [71]. In fact, Sakwinska et al. reported that only vaginal delivered and fully breastfed infants had gut microbiota dominated by *Bifidobacteria* [62]. 

Finally, there are several potential preventive intervention strategies to restore the gut microbiota after C-section [72]. The intervention could be focused on maternal administration of probiotics and prebiotics during gestation. There is a great interest about “seeding approaches” as “vaginal seeding” to reverse the effects of C-section delivery mode on the microbiome in early life, but at the same time there are critical voices concerned about safety and efficacy of this practice [56,72]. In addition, the intervention could concentrate on the neonate using “seeding” methods such as encouraging breastfeeding instead of formula feeding, or the use of infant enriched formulas. In this sense, supplementation with symbiotic, the combination of synergistic pre- and probiotics, might offer an innovative strategy to re-establish the delayed colonization of *Bifidobacterium* spp. in C-section-delivered children [73].

## 4. Microbiome and the Type of Feeding 

Maternal diet establishes long-lasting effects on offspring gut microbial composition, which may have important clinical implications [74,75]. Complex interactions between breast milk cytokines and microbiota guide the microbiological, immunological, and metabolic programming of infants’ health, which may explain the higher risk of obesity in infants with overweight and excessive weight gain mothers [76]. In addition, data supporting the notion of bacterial translocation from the maternal gut to extra-intestinal sites during pregnancy are emerging and potentially explain the presence of bacteria in breast milk [28].

Some authors have reported changes in meconium microbiota when delaying the collection of meconium samples by one day, supporting that the type of feeding or the environment has an influence after the birth, which may be more determinant to establish the intestinal microbiome during childhood [53]. Breast milk has been recognized as the gold standard for human nutrition [77]. The type of feeding has an important impact on gut microbial composition in preterm infants. In preterm infants, breast milk has been associated with improved growth and cognitive development [78] and a reduced risk of necrotizing enterocolitis and late sepsis onset [76,79,80]. Occasionally, the absence of mother’s own milk (MOM) requires the use of donated human milk (DHM). A prospective cohort study has been launched to determine the impact of DHM upon preterm gut microbiota admitted in a neonatal intensive care unit. Despite the high variability of DHMs, no differences in microbial diversity and richness were found, although feeding type significantly influenced the preterm microbiota composition and predictive functional profiles. Inferred metagenomic analyses showed higher presence of *Bifidobacterium* in the MOM, a genus related to enrichment in the glycan biosynthesis and metabolism pathway, as well as an unclassified *Enterobacteriaceae* and lower unclassified *Clostridiaceae* compared with the DHM or in the formula fed groups. After adjusting for gender, postnatal age, weight, and gestational age, the diversity of gut microbiota increased over time and was constantly higher in infants fed their MOM relative to infants with other types of feeding. In addition, DHM favors an intestinal microbiome more similar to MOM despite the differences between MOM and DHM [81]. Preterm infants are prone to develop free radical-associated conditions [82] that may be influenced by the microbiota. In a recent study, urine oxidative stress biomarkers such as 8-hydroxy-deoxyguanosine (8OHdG/2dG), orto-tyrosine, and F2 isoprostanes, neuroprostanes, neurofurans, and di-homo-isoprostanes were longitudinally measured in preterm infants fed either MOM or DHM using validated mass spectrometry techniques. No significant differences for any of the markers studied were found between preterm babies fed MOM or DHM [83]. However, exfoliated epithelial intestinal cells transcriptome of preterm infants fed their MOM or a DHM induced a differential gene expression of specific genes which may contribute to a more efficient antioxidant response in the postnatal period [84]. Therefore, using DHM could have potential long-term benefits on intestinal functionality, the immune system, and metabolism [85,86,87]. However, available pasteurization methods cause changes that may blunt many of the positive aspects derived from the use of MOM [88,89,90]. Further studies are needed to understand the complex links between microbiome and breastfeeding, its impact on health programming, and to develop sensitive methods capable of providing human milk as similar as possible to their MOM, when the latter is not available.

## 5. Microbiome in Pathological and Adverse Pregnancy Outcomes

Some studies have compared the fetal and mother microbiome in relation to adverse outcomes such as prematurity or low birth-weight without reaching firm conclusions. Ardissone et al. [91] compared the meconium microbiome in newborn before and after 33 weeks of gestation and concluded that *Enterococcus* and *Enterobacter* negatively correlated with gestational age, and *Lactobacillus* and *Phortorhabdus* were more abundant in newborns with less than 33 weeks of gestation. They indicated that the composition of the microbiome may be involved in the inflammatory response that leads to premature birth more than the colonization alone. Specifically, preterm subjects with severe chorioamnionitis had higher abundance of *Ureaplasma parvum*, *Fusobacterium nucleatum*, and *Streptococcus agalactiae* [16]. The placental microbiome varies as a consequence of an excess of gestational weight gain, but is not related to obesity among women with spontaneous preterm birth. Indeed, this placental dysbiosis affects different bacterially encoded metabolic pathways that may be related to pregnancy outcomes [92]. Furthermore, it has been reported high abundance of *Burkholderia*, *Actinomycetales*, and *Alphaproteobacteria* in placental samples from gravidae delivered preterm, and of *Streptococcus* and *Acinetobacter* in placental samples from patients with a history of antepartum urinary infection. In contrast, *Paenibacillus* predominated in term placental specimens [15]. Other authors have proposed that the fetal intestinal microbiota derives from swallowed amniotic fluid, and that they may trigger an inflammatory response which leads to premature birth [91]. Considering that some *Lactobacillus* strains may possess potential anti-inflammatory activities, and could regulate blood glucose levels in diabetic humans [93], the low abundance of *Lactobacillus* in placentas of low birth weight neonates reported by Zheng et al. [94] might be related to a pro-inflammatory status in these pregnancies. Thus, the higher sensitivity of fetal intestinal tissue to inflammatory stimuli may induce labor due to an immune-mediated reaction. However, as mentioned previously, the presence of placental microbiota is under discussion due to methodological doubts, and these data have to be discussed with caution.

Finally, a number of bacteria, viruses, and protozoa infections have been associated with pregnancy complications. Liu et al [95] analyzed the gut microbiome in pregnant women affected by preeclampsia. They showed an overall increase in pathogenic bacteria such as *Clostridium perfringens* and *Bulleidia moorei* and a reduction in probiotic bacteria *Coprococcus catus*. A correlation between periodontitis and the risk of spontaneous abortion or miscarriage has also been described [96]. More well-controlled studies should be carried out in order to identify interactions between pregnancy microbiome and mother and children health which might help to predict gestational and newborn complications and search for new therapeutic targets in adverse obstetrical conditions.

## 6. Microbiome and Obese Pregnancy

Epidemiological evidence shows that 50% of women in childbearing age and 20%–25% of pregnant women in Europe can be affected by overweight or obesity [97], increasing the cardiometabolic risk in mothers [98] and the susceptibility to metabolic diseases in offspring [99,100,101,102]. Pregnancy-associated changes are different in overweight or obese women compared to normal-weight pregnant women. Overweight pregnant women show a reduction in the number of *Bifidobacterium* and *Bacteroides,* and an increase in the number of *Staphylococcus*, *Enterobacteriaceae*, and *Escherichia coli* [11]. Additionally, higher levels of *Staphylococcus* and *Akkermansia muciniphila,* and lower levels of *Bifidobacterium* were detected in women with excessive weight gain during pregnancy as compared to normal-weight ones [76]. Consequently, this altered maternal microbiome will contribute to shape an altered composition of the offspring’s microbiome [103,104] and thus influence their future health. 

Vaginal-born neonates from overweight or obese mothers show increased numbers of *Bacteroides* and depleted in *Enterococcus*, *Acinetobacter*, *Pseudomonas*, and *Hydrogenophilus* [104]. When specifically examining phyla level relative taxonomic abundance among preterm women by virtue of maternal weight gain, other authors have reported an appreciable and significant increased abundance of *Firmicutes*, *Actinobacteria*, and *Cyanobacteria*, and decreased relative abundance of *Proteobacteria* [92]. Furthermore, this altered maternal microbiota composition may be transferred from mother to fetus during the prenatal period [94] and through lactation [105]. 

In addition, gut microbiota can induce obesity in children by several mechanisms. For example, lower amounts of *Bifidobacteria* can affect weight gain in infants through mucosal host-microbe crosstalk, and immune and inflammatory dysregulation. Moreover, higher presence of *Bacteroides*, *Clostridium*, and *Staphylococcus* can stimulate greater energy extraction from food, combined with a reduced control of inflammation during the first six months of life in infants of overweight mothers [12]. These first months of life are of great importance since rapid weight gain during this period is associated with an increased risk of obesity during childhood, and this influence is even more important than the birth weight [106].

## 7. Microbiome in Critical Ill Children

Critical illness itself or its treatment can influence the composition of microbiota [107,108]. Although broad-spectrum antibiotics are probably the factor which further alters its composition, other factors can alter the ecosystem in which develops the microbiota, such as enteral or parenteral feeding, drugs administration, disease co-morbidities, central venous catheters, or intubation and mechanical ventilation. These studies have shown that the intestinal microbiota of critical patients has low diversity, with a shortage of key commensal bacteria and overgrowth of pathogenic bacteria such as *Clostridium difficile,* and some species of *Enteococcus, Escherichia* and *Shigella* [107,109,110,111]. In addition, the microbiota changes throughout the stay in the intensive care units (ICU) [112], and the possibility of pathogenic colonization increases with the time of stay in the unit. 

To our best knowledge, only one study has analyzed the microbiota in children in a pediatric ICU (PICU) [113]. These authors found that the skin, oral, and fecal microbiota differs sharply from critically ill children compared with healthy children and adults. They reported a PICU-associated dysbiosis with less alpha-diversity, different composition (beta-diversity), and the loss of body site-specificity, increasing the abundance of nosocomial pathogens across all body sites and reducing gut commensals such as *Faecalibacterium* [113]. A number of studies have shown an association between the microbiota and the immune function [114], the systemic inflammation [115], the metabolism of nutrients [116], the function of the central nervous system [117], the circadian rhythm [118], and the digestive system [119]. Therefore, PICU-associated dysbiosis may contribute to malnutrition, nosocomial infection, neurocognitive alteration, organ dysfunction, and sepsis associated to critical illness [113], and may also have an effect on the lung, the brain, and the kidneys [107].

Critically ill patient conditions may contribute to changes in the oropharynx microbiota, such as the increase of *Klebsiella* or *Pseudomonas* proliferation. On one hand, sedation and endotracheal intubation decrease mucociliary clearance and cough, reducing the elimination of microorganisms. On the other hand, mechanical ventilation, pneumonia, and acute respiratory distress syndrome (ARDS) favor alveolar edema, increasing the amount of nutrients available and decreasing the amount of oxygen in some areas. These facts stimulate bacterial proliferation [120], and increase the risk of nosocomial infection and ARDS [121]. 

In addition to the critical patients, associated dysbiosis, hypoperfusion, and reperfusion of the intestinal wall produce an intense inflammation of the digestive mucosa which alters the gradient of oxygen concentration and increases the concentration of nitrates favoring the growth of pathogenic flora. Furthermore, the slowing down of intestinal transit, frequent drugs (sedatives, opioids, catecholamines), and the alteration of the mechanisms of microbial elimination (decreased production of bile salts and IgA, pharmacological alkalinization of pH, etc.) may also influence the alteration of the digestive functions [122]. Freedberg et al. observed that colonization by some microorganisms prior to admission in ICU was associated with increased risk of infection by that same germ, and subsequently increased mortality [123]. This fact indicates that the gastrointestinal microbiome can help stratification and early identification of the risk of ICU patient complications.

## 8. Microbiome and Sepsis in the Newborn

The modification of the normal microbiota pattern can contribute to the development of a systemic inflammatory response with increased cytokine production, sepsis, multi-organ failure, and morbi-mortality [107,109,110,111]. In spite of variation in net incidence, neonatal sepsis remains one of the leading causes of preventable neonatal morbidity and mortality throughout the world. The main agents responsible for sepsis are group B *Streptococcus* (GBS), *Escherichia coli*, and coagulase-negative *Staphylococci* (CONS) [124]. However, this scenario may be modified depending on the use of antibiotics and/or the implementation of non-culture diagnostic techniques [125].

In recent years, there has been growing interest in the role of commensal bacteria in an individual´s susceptibility to infection. A few studies have evaluated the maternal vaginal microbiota in relation to GBS carrier status. Although it seems that some specific taxa might be associated with the presence of GBS [126], there is no apparent parallel reduction of the predominant commensal bacteria *Lactobacilli* [127]. Indirect evidence suggests that the neonatal gut microbiome might be of relevance in GBS infection, since different colonizing species have been found in the stool of infants from GBS positive and negative mothers, while the protective effect of pre and probiotics has also been suggested [127].

It seems that gastrointestinal microbiota might induce an increase in permeability, modulating gut and systemic immune response, and decreasing the tight junction integrity [128]. As a consequence, intestinal bacteria can promote the systemic inflammatory response syndrome, facilitate bacterial translocation, and cause late-onset sepsis and necrotizing enterocolitis, especially affecting premature neonates. Most, but not all, of the evidence suggests that premature newborns with low microbiome gut diversity, or with predominance of *Staphylococcus, Firmicutes*, and *Proteobacteria* are associated with increased risk for late-onset sepsis compared to those premature infants at lower risk [129]. Furthermore, gut colonization with *Bifidobacterium* and increased presence of prebiotic oligosaccharides in feces, has been related to less disruption of the mucosal barrier and gut epithelial translocation, providing an improved gut development and protection [130]. It remains unclear if invasion of the bloodstream during sepsis is caused by the same microorganisms identified in stool [131] or by others [129], in which case the gut microbiota would act as a facilitating mechanism by interfering with the gut barrier or intestinal immune function. Further studies are needed to tease out if the differences observed in gut colonization in ICU patients predispose to sepsis or if they respond to other factors such as the diet, site differences in initiating and advancing feeds, breastfeeding, the use of antibiotics, or interpatient transmission within the neonatal intensive care units [131].

## 9. Microbiome and Allergic Conditions

Allergy disorders represent an important global health burden with an increasing prevalence in infants and children, mainly as food allergies, atopic eczema [132], and respiratory pathologies such as rhinitis [133] or asthma [134]. Their causes are multifactorial and contemplate interactions between genetic, environmental, and socioeconomic factors leading to different symptoms or phenotypes [135]. Among this heterogeneity, a restricted microbial exposure at early life seems to play an important role influencing allergic diseases, and asthma onset [136]. 

### 9.1. Gut Microbiome and Atopy

Eczema or atopic dermatitis (AD) is the first typical allergic manifestation in newborns [137]. A recent study has reported a high proportion of *Faecalibacterium prausnitzii* on the gut microbiome from AD subjects. The presence of these bacteria is lower in Crohn’s disease patients, as well as anti-inflammatory fecal bacteria metabolites [138]. Besides, it has been shown that infants with AD improved their symptomatology when the abundance of fecal *Coprococcus eutactus*, a butyrate-producing bacterium, is increased [139]. Consequently, it has been proposed that dysbiotic gut microbiota and subsequent dysregulation of the gut inflammation may promote an aberrant Th2-type immune response to allergens altering the epithelial barrier in AD skin [140].

### 9.2. Gut Microbiome and Food Allergy

Available literature on animal models suggests that gut microbiome may have an important role in the susceptibility to food sensitization and food allergy, mainly at early stages of life [141]. Chen et al. [142] recently showed both lower microbiota alpha-diversity and altered gut microbiota composition (an increased number of *Firmicutes* in detriment of *Bacteroidetes*) in children with food sensitization in early life compared with children without these conditions. Among the causes, the increasing use of antibiotics both in humans and in agriculture, and the lower intake of dietary fiber may have an impact on these situations [143].

### 9.3. Gut Microbiome and Asthma

Allergies are the strongest risk factors for childhood asthma in Western countries [144], but the relationship between asthma and the microbiota is not clear. Although it seems that the diversity of the gut microbiota in infancy is even more determinant for asthma onset than the prevalence of specific bacterial taxa, it has been suggested that there might be specific important bacterial species related to the prevention of asthma, and that gut microbial diversity during the first month of life may be the most important factor associated with asthma development at school age than with other allergic manifestations [136]. In addition, another study has indicated that the neonatal gut microbiota influences susceptibility to childhood allergic asthma via alterations in the gut microenvironment that modulates CD4+ T-cell proliferation and functions. These authors have observed a characteristic depletion of dihomo-γ-linoleate, a precursor of anti-inflammatory ω-3 polyunsaturated fatty acid and prostaglandins that may be related [145].

As described previously, different factors have been associated with infant microbiome and the risk of asthma, such as furry pets exposure [146], gestational age, the mode of delivery (vaginal vs. C-section), and antibiotic treatment (direct vs. indirect via mother) among others [147,148]. However, there is no doubt that a key issue is the type of feeding. A systematic review addressing the effect of breastfeeding in the development of asthma concluded that children who were breastfed for a longer time during the first two years of life had a lower risk of developing asthma, and this effect could be mediated by an adequate and early shaping of the gut microbiota [149,150], although whether the dysbiotic microbiota is the cause or the consequence of atopic and allergic diseases is still unknown [140]. Besides, interventional studies have suggested that pre- and probiotics could prevent or down-regulate the severity of some diseases, such as asthma or allergies, but the biological mechanisms, as well as the best taxa or type of intervention, require further research [151]. 

## 10. Microbiome and Infection in Infants

The role of microbiome diversity and its variations in the incidence and susceptibility to infection has also aroused great interest beyond the neonatal period. In view of the interaction between the microbiota and the immune system, the implications are probably major and remain challenging, but for some authors, is even more attractive the idea of its usefulness as a diagnostic tool, a preventive strategy, or even a therapeutic target. As described in the neonatal period, in most infectious diseases scenarios, a decrease in alpha and beta diversity of the microbiota seems to be present. Regarding respiratory infections, diversity of the oropharyngeal and nasopharyngeal microbiota in children with pneumonia was lower compared with healthy controls. Furthermore, a correlation between the presence of certain taxa in sputum and the clinical course of community acquired pneumonia has been described [152,153]. 

HIV infected children present reduced gastrointestinal microbial diversity [154]. Modulation of the intestinal microbiome through nutritional supplementation, with the aim of decreasing bacterial permeability, has been attempted in the context of HIV infection with scarce success [155,156]. In addition, the microbiome has been suggested to impact the risk of different infectious diseases. Both vaginal and penile microbiotas modify the risk of sexual acquisition of HIV, due to their influence on inflammatory pathways and metabolization of antiretroviral drugs [157,158]. Recent studies have shown how an altered vaginal microbioma increases the risk of vertical transmission of HIV [159]. These studies beautifully exemplify the potential influence of the microbiome on the risk of infections, as well as its implications in pharmacokinetics modulating bacterial metabolism.

Finally, based on the potential role of the gut microbiota as a modulator of the immune function, attempts of supplementation with pre and probiotics have also been carried out. Two randomized controlled trials have analyzed the impact of probiotic supplementation on children with acute gastroenteritis without proving any beneficial clinical outcome [160,161]. Supplementation with prebiotics or probiotics may also enhance vaccine response and thus becomes a new tool for the improvement of vaccine efficacy [162]. However, results have been controversial in this field and warrant further investigation. The evidence for a beneficial effect of probiotics on vaccine response was strongest for oral vaccinations and for parenteral influenza vaccination, and depended on the choice of probiotic, strain, dose, viability, purity, and the time and duration of administration [163]. 

## 11. Conclusions

There are many data confirming the interaction of microbiota in pregnancy and in the newborn period, on the establishment of labor, children growth and development, and susceptibility to infections and diseases. However, most studies are descriptive and entangling factors influencing the human microbiome such as the age, race, type of feeding, mother’s diet, and antibiotics treatments is challenging. Whatever it is, what is clear is that a number of microbiota-derived substances may easily reach the bloodstream, and impact human metabolism. 

Recent advances in genome sequencing technologies, metabolomics, proteomics, transcriptomics, and bioinformatics will enable researchers to explore the fascinating field of the microbiota and, in particular, its functions at a more detailed level. Therefore, larger and prospective studies are needed to characterize the evolution of the microbiota during different conditions and its influence on healthy and pathological pregnancies, on labor onset, and on the perinatal period, in order to promote the development of new preventive, diagnostic, and therapeutic tools.
